# Timing-specific efficacy of antiviral baloxavir and anti-inflammatory oclacitinib monotherapies, and the benefits of their combination in treating influenza in mice

**DOI:** 10.3389/fmicb.2026.1741128

**Published:** 2026-03-16

**Authors:** Yang Yu, Lefang Jiang, Jiaxin Ke, Xiaoqin Lian, Yarou Gao, Xingjian Zhu, Mingxin Zhang, Huixia Wu, Xulin Chen

**Affiliations:** 1 Department of Immunology and Microbiology, College of Life Science and Technology, Institute of Medical Microbiology, Jinan University, Guangzhou, China; 2 Kunming National High-Level Biosafety Research Center for Non-Human Primates, Center for Biosafety Mega-Science, Kunming Institute of Zoology, Chinese Academy of Sciences, Kunming, Yunnan, China; 3 Department of Rheumatology, First Affiliated Hospital, Jinan University, Guangzhou, China

**Keywords:** anti-inflammatory, antiviral, baloxavir, combination therapy, influenza virus, Janus kinase (JAK) inhibitors, oclacitinib

## Abstract

Excessive inflammation from uncontrolled pro-inflammatory cytokine release is a leading cause of mortality in influenza virus infections. Anti-inflammatory therapies, particularly Janus kinase (JAK) inhibitors, have demonstrated protective effects in murine models against lethal influenza virus infections, particularly during the later stages of infection. This study investigates the potential synergy of combining antiviral and anti-inflammatory medications to extend the treatment window for influenza. We assessed the *in vivo* therapeutic windows of the antiviral baloxavir and the JAK inhibitor oclacitinib, both as monotherapies and in combination. Baloxavir proved highly effective when administered early during influenza infections; however, its efficacy rapidly declined with administration 1 day post-infection (p.i.) and was nearly absent by 2 days. In contrast, administration of oclacitinib at the mid-stage of disease effectively protected mice from lethal infections. The combination therapy of baloxavir and oclacitinib significantly extended the therapeutic window compared to monotherapies alone. The data indicate that the combination of baloxavir and oclacitinib not only extends the therapeutic window for both agents but also presents a promising new approach for treating influenza virus infections. These findings highlight the potential benefits of combining antiviral and anti-inflammatory therapies to enhance patient outcomes.

## 1 Introduction

Influenza virus infection remains a significant public health challenge, contributing to an average of 700,000 deaths due to respiratory and cardiovascular illnesses annually (Paget et al., 2019; Chaves et al., 2023). Several major influenza pandemics have occurred over the past five decades, including the Hong Kong Flu (1968–1969) (Viboud et al., 2005), Russian Flu (1977–1978) (Kung et al., 1978), and H1N1 Pandemic (2009–2010) (Zarocostas, 2009), leading to millions of fatalities. High-risk populations, such as the elderly, young children, and those with underlying health conditions, remain particularly vulnerable (Thompson et al., 2009; Nair et al., 2011).

Influenza can cause severe illness by directly damaging the respiratory epithelium and triggering an excessive immune response, known as a “cytokine storm,” leading to lung tissue damage and respiratory failure (Kalil and Thomas, 2019). Furthermore, it increases susceptibility to secondary bacterial infections and worsens outcomes, especially in individuals with chronic conditions, such as asthma, chronic obstructive pulmonary disease (COPD), heart disease, or diabetes (Thompson et al., 2009; Nair et al., 2011). Timely antiviral and anti-inflammatory therapies are crucial for managing severe influenza infections.

Current antiviral therapies for influenza include several medications effective for both prevention and treatment. Oseltamivir, zanamivir, and peramivir target viral neuraminidase, impeding the release of virions from the infected cells. These neuraminidase inhibitors are most efficacious when initiated within 48 h of symptom onset (Gubareva et al., 2000; Chan and Hui, 2023). Baloxavir marboxil, a novel antiviral administered as a single dose, targets the viral cap-dependent endonuclease, an enzyme pivotal in the process of “cap snatching,” thereby inhibiting viral mRNA synthesis (Tejus et al., 2022; Chan and Hui, 2023). In addition to the emergence of drug-resistant strains, the narrow therapeutic windows of all direct-acting antiviral agents pose challenges to their clinical application.

Anti-inflammatory therapy is crucial for managing the excessive immune response to influenza, especially when antiviral treatment is delayed. Anti-inflammatory medicines are critical for reducing excessive immune responses and preventing complications such as ARDS and secondary bacterial infections, which aid recovery (Davidson, 2018; Li et al., 2018). However, conventional anti-inflammatory drugs such as corticosteroids may worsen outcomes (Carey et al., 2010; Brun-Buisson et al., 2011; Kim et al., 2011). Current research has focused on identifying effective anti-inflammatory agents, such as statins and COX-2 inhibitors (Ramos and Fernandez-Sesma, 2015; Rodrigo et al., 2016; Lim et al., 2020).

More recently, several reports, including ours, found that protein tyrosine kinase inhibitors targeting MAPK and JAK showed promising results in protecting mice infected with lethal influenza viruses (Borgeling et al., 2014; Chen et al., 2019; Wang et al., 2020; Schnepf et al., 2021; Yu et al., 2024; Chen et al., 2025). However, the timing of administration is critical. Early use may enhance viral replication and compromise efficacy, whereas later administration can improve outcomes (Chen et al., 2019; Yu et al., 2024). This highlights the importance of exercising caution when using anti-inflammatory therapies.

Currently, specific immunomodulatory agents for treating severe influenza virus infections are not available in clinical practice. Given the narrow therapeutic windows of both antiviral and anti-inflammatory agents, the exploration of novel drug combinations encompassing both is warranted in animal models and clinical settings to expand the therapeutic windows of monotherapies using either antiviral or anti-inflammatory agents alone.

In this study, we evaluated the effectiveness of combining the antiviral drug baloxavir with the anti-inflammatory agent oclacitinib in a mouse model of influenza. Our goal was to determine whether this combination was more effective and with a wider therapeutic window than individual monotherapies. We found that baloxavir works well when administered early, whereas oclacitinib is beneficial in the mid-stages of infection. However, the early use of oclacitinib worsens the disease by increasing viral replication. The combined treatment enhances the therapeutic effects of both agents, offering a promising new strategy for managing influenza and related viral infections with inflammatory complications.

## 2 Materials and methods

### 2.1 Cell lines, animals, and virus strains

Madin-Darby canine kidney (MDCK) cells (ATCC CCL-34™) were cultured in Dulbecco’s modified Eagle’s medium (DMEM; Gibco, Carlsbad, CA, United States) supplemented with 10% fetal bovine serum (Gibco). The influenza virus strain A/Puerto Rico/8/1934 (H1N1) was obtained from the Wuhan Institute of Virology, Chinese Academy of Sciences. Virus titers were determined using 50% tissue culture infective dose (TCID50) assays in MDCK cells. Ten-day-old embryonic chicken eggs were obtained from Guangdong Wens Dahuanong Biotechnology Co., Ltd., Guangdong, China. Six-week-old BALB/c mice were acquired from Guangdong Medical Laboratory Animal Center (GDMLAC) and maintained under specific pathogen-free conditions.

### 2.2 Chemicals

Both oclacitinib (T6914) and baloxavir (T6195) were purchased from TargetMol (Shanghai, China). The compounds were initially dissolved in a 5% dimethyl sulfoxide (DMSO) solution for storage. Subsequently, they were diluted to the required final concentrations using a 0.9% sodium chloride solution containing 0.5% carboxymethyl cellulose sodium (CMC-Na), 30% polyethylene glycol 300 (PEG300), and 10% tween 80.

### 2.3 Enzyme-linked immunosorbent assay (ELISA)

Mouse IL-10 (Cat. No. 431414), IL-6 (Cat. No. 431304), and TNF-α (Cat. No. 430901) ELISA kits were obtained from BioLegend (San Diego, CA). Mouse IL-8 (CME0008), MCP-1 (CME0046), and IP-10 (CME0051) ELISA kits were acquired from 4A Biotech (Beijing, China). The mouse CCL3/MIP-1α (EK261/2-96) ELISA kit was procured from Multi Sciences (Hangzhou, China). The mouse CCL5/Rantes (BSEM-048-96T) ELISA kit was obtained from Biosharp (Hefei, China). The ELISA was performed according to the protocol specified for each kit.

### 2.4 Animal experiments

All the mice used in the animal experiments were 6-week-old female BALB/c mice. The mice were anesthetized with inhaled isoflurane (3% in oxygen) and then intranasally infected with a mouse-adapted strain of the influenza virus A/Puerto Rico/8/1934 (H1N1) at a dose of 300 TCID50 in 50 μL. Mice in the mock group received an equivalent volume of phosphate-buffered saline (PBS) without viral infection.

In the oclacitinib monotherapy study, oclacitinib was dissolved in DMSO and diluted in 0.9% sodium chloride containing 0.5% CMC-Na, 30% PEG300, and 10% tween 80, with a final DMSO concentration of 5%. Oclacitinib was administered orally at a dosage of 20 mg/kg/day, twice daily, for five consecutive days. For baloxavir monotherapy, baloxavir was dissolved in DMSO and diluted in 0.9% sodium chloride containing 0.5% CMC-Na, 30% PEG300, and 10% tween 80, with a final DMSO concentration of 5%. Baloxavir was administered orally once at the indicated time points during the experiment.

In the combination therapy, both agents were administered simultaneously. baloxavir was given at 10 mg/kg/day once, while oclacitinib was administered at 20 mg/kg/day twice daily for 5 days.

Following infection, all mice were monitored daily for survival, weight loss, and clinical signs of illness, including lethargy, piloerection, ruffled fur, hunched posture, rapid, shallow breathing, and audible crackling. Clinical scores ranging from 0 (no symptoms) to 5 (death or moribund) were recorded daily for each mouse, as in previous studies (Yu et al., 2024).

On day 7 p.i., both mock- and infected-mice were sacrificed. Their tracheas and lungs were washed 3 times with 1 ml of PBS containing 0.1% BSA. After centrifugation for 10 minutes at 1,000 × g and 4°C, bronchoalveolar lavage fluid (BALF) was collected for further analysis.

### 2.5 Preparation of BALF

Mice were euthanized with cervical dislocation and secured onto a surgical plate, followed by disinfection of the cervical region using 75% ethanol. An incision was made in the cervical skin using scissors to expose the trachea. Subsequently, a 0.2 cm incision was created on the trachea with surgical scissors, and a gavage needle was carefully inserted into it. The trachea was ligated around the needle using cotton thread to secure its position. Subsequently, a sterile PBS solution was gently injected into the lung using a 1 mL syringe. Before careful aspiration, the mouse’s thorax was manipulated to facilitate uniform distribution of the solution. Following removal of the syringe from the needle, the lavage fluid obtained from this procedure was transferred to an ice-cooled 1.5 mL tube for further processing or stored at −80°C for subsequent analysis. Finally, cell pellets obtained after centrifugation of lavage fluid for 10 min at 1,000 × g and 4°C were resuspended and stained for subsequent flow cytometric analysis.

### 2.6 Lung tissue histopathology

Hematoxylin and eosin staining was performed as previously described (Yu et al., 2024). All mouse lung tissues were fixed in 4% paraformaldehyde overnight, embedded in paraffin, and sectioned into 4 μm-thick sections. Subsequently, all the sections were stained with hematoxylin solution, treated with hematoxylin differentiation solution and hematoxylin Scott tap bluing, and rinsed with tap water. The sections were dehydrated in 85% ethanol for 5 min, then in 95% ethanol for 5 min, and finally stained with eosin for 5 min. Following dehydration, sections were mounted in a neutral mounting medium. Each section was examined microscopically, and the slides were randomly assessed and analyzed for tissue damage, necrosis, and inflammatory cellular infiltration.

### 2.7 Statistical analyses

Quantitative data for ELISA, body weight, clinical score, and survival rate of animal experiments were generated using the Prism software (version 8.0; GraphPad Software, San Diego, CA, United States). Data are presented as mean ± SEM for each point. Differences in ELISA results, body weight, and clinical scores were statistically analyzed using Student’s *t*-test. The significance of differences in survival rates was compared using the log-rank (Mantel-Cox) test, as described in the figure legends. Statistical significance was set at *P* < 0.05; significant results are indicated by (*). *p* < 0.01, highly significant (**). *p* < 0.001, very highly significant (***).

## 3 Results

### 3.1 Baloxavir monotherapy protects mice infected with the lethal influenza virus when administered early

The clinical studies demonstrated that a single dose of baloxavir was superior to placebo in alleviating influenza symptoms and more effective than both oseltamivir and placebo in reducing viral load 1 day after initiation of the trial regimen in patients with uncomplicated influenza (Hayden et al., 2018). In this study, a series of early and delayed administrations was used to evaluate the therapeutic window in mice infected with a lethal strain of influenza. Six groups of 10 mice received either a placebo or 10 mg/kg/day baloxavir during infection or at 1, 2, 3, or 4 days post-infection (dpi). The mock-infected control group received phosphate-buffered saline (PBS) as a control treatment. Mice were monitored for survival, body weight loss, and clinical signs over 21 days, with scores ranging from 0 (no symptoms) to 5 (death), recorded daily (Yu et al., 2024).

**FIGURE 1:**
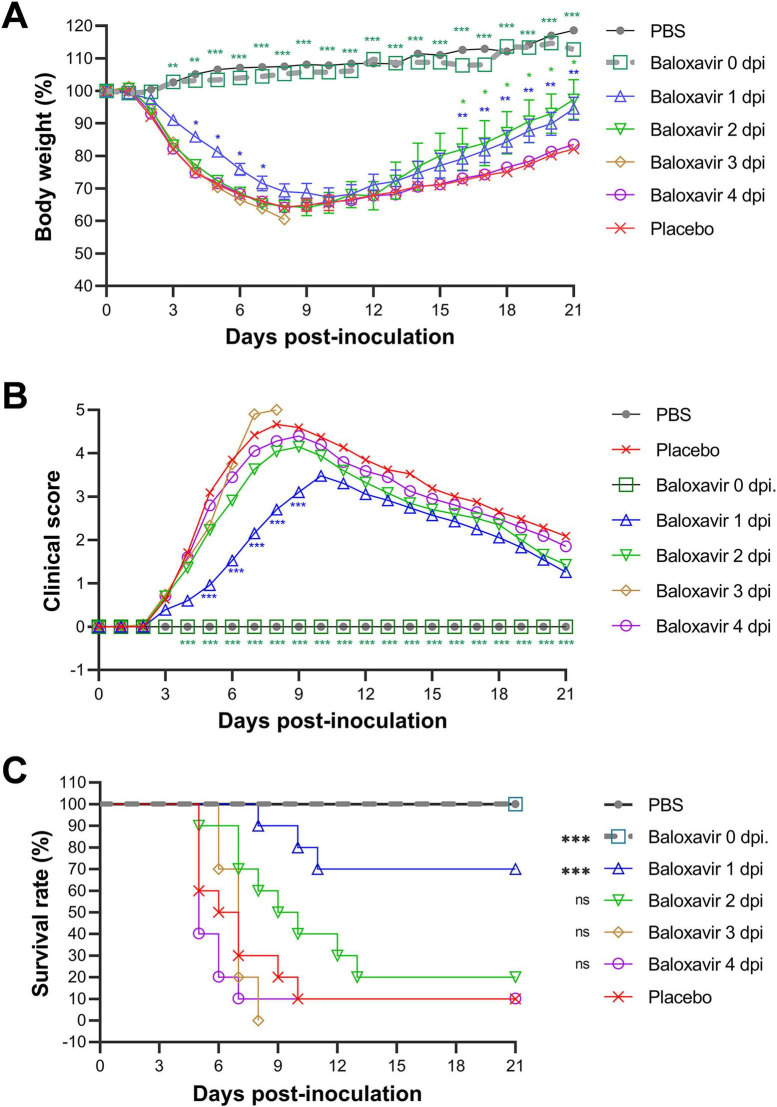
Assessment of the therapeutic window for monotherapy with antiviral baloxavir in treating lethal influenza in a murine model. Five groups of mice were infected with 300 TCID50 of PR8 virus (*n* = 10 mice per group) and subsequently treated with baloxavir (10 mg/kg) at various time points: 0, 1, 2, 3, or 4 days post-infection (dpi). One group received a placebo at day zero to serve as a treatment control, while the other group underwent mock infection using an equal volume of phosphate-buffered saline (PBS) to act as a non-infection control. Body weight loss (%) **(A)**, clinical score **(B)**, and survival rate (%) **(C)** were recorded daily for 21 days. Statistical analyses for body weight loss **(A)** and clinical score **(B)** were conducted using two-way ANOVA with Sidak’s *post-hoc* test, while the log-rank test was employed to assess survival rates **(C)**. The significance levels are indicated as follows: *p < 0.05, **p < 0.01, ***p < 0.001; ns denotes no significance.

As shown in Figure 1A, body weights increased in mock-infected or PR8-infected and baloxavir-treated (starting at 0 dpi) mice, whereas mice in the other four groups treated at a later time exhibited faster weight loss. Mice treated starting at 1 dpi showed a milder weight decrease than those treated at 2, 3, and 4 dpi. In terms of the clinical score, infected mice treated with baloxavir from 0 dpi onward exhibited a similar health status to mock-infected mice. Mice treated with baloxavir at 1 dpi displayed mild clinical symptoms, with a peak score of 3.48 at 10 dpi. In comparison, the peak score for placebo-treated mice was 4.57 (day 8), while the peak score for baloxavir-treated mice was 4.35 (day 9), 5 (day 8), and 4.6 (day 8), respectively, for treatments starting at 2–4 dpi (Figure 1B).

Notably, the survival rate of mice treated with baloxavir at 1 dpi was 70% (7/10), which was significantly higher (*p* < 0.001) than that of the placebo-treated mice (10%, 1/10). Moreover, all mice survived the lethal dose of the influenza virus immediately after infection (100%, *p* < 0.001). However, this protective effect was not significant when mice were treated 2 dpi or later (ns, *p* > 0.05) (Figure 1C).

The present findings suggest that baloxavir demonstrates superior efficacy when administered within 24 h of the onset of viral infection. Administering baloxavir after the onset of symptoms (at 2 dpi) was ineffective in treating mice infected with a lethal influenza virus. This conclusion is further supported by data from viral titers, cytokine levels, and histopathological analyses.

**FIGURE 2:**
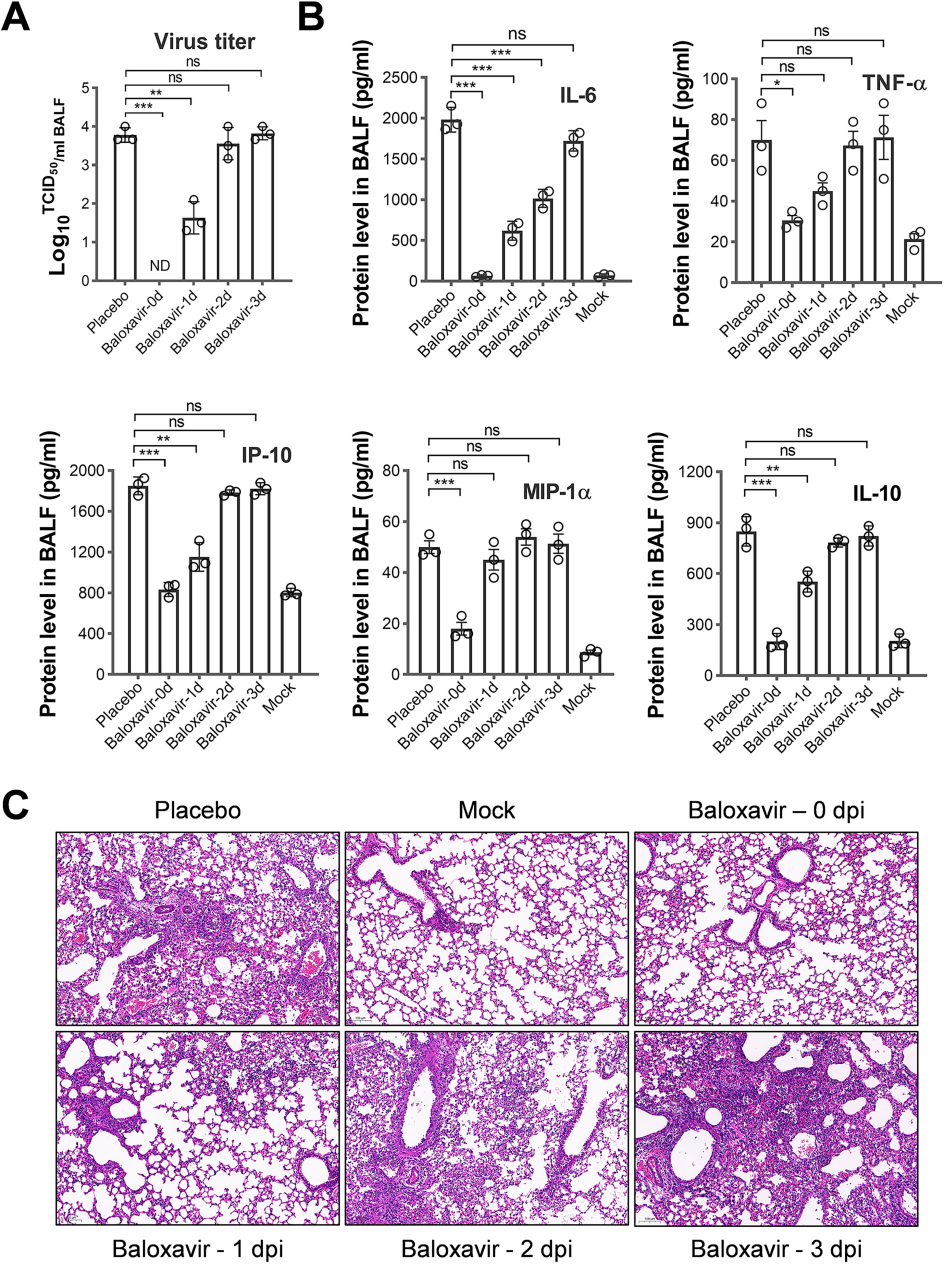
Virus production, cytokine/chemokine levels, and lung damage assessment in mice infected with lethal influenza and treated with baloxavir at various time points. Four groups of mice were infected with 300 TCID50 PR8 virus (3 mice per group) and subsequently treated with baloxavir (10 mg/kg) at 0, 1, 2, or 3 dpi. The control group received placebo treatment at 0 dpi, following infection with 300 TCID50 of the PR8 virus. Additionally, the mock-infected group was administered an equivalent volume of phosphate-buffered saline (PBS) to serve as a non-infection control. All mice were euthanized with cervical dislocation on day 7 p.i., and BALFs were collected to assess cytokine levels, progeny viral titers, and lung tissue histopathology. **(A)** Viral titers in BALFs obtained on day 7 p.i. were evaluated by TCID50 assay using Madin-Darby canine kidney (MDCK) cells (3 mice per group). **(B)** The protein concentrations of pro-inflammatory cytokines and chemokines in BALFs corresponding to the various baloxavir treatment groups were quantified using enzyme-linked immunosorbent assay (ELISA) on day 7 p.i. **(C)** Lung tissue sections from mice treated with baloxavir, placebo, and PBS were stained with hematoxylin and eosin, with a scale bar representing 100 μm. Data are presented as mean ± standard error of the mean (SEM). Statistical significance in panels **(A,B)** was determined using unpaired Student’s *t*-test. The significance levels are denoted as follows: *p < 0.05, **p < 0.01, ***p < 0.001; ns indicates no significance.

As shown in Figure 2A, when baloxavir was administered at the time of virus inoculation, virus titers in bronchoalveolar lavage fluid (BALF) remained consistently low, reaching undetectable levels. Conversely, initiating baloxavir treatment at 1 dpi reduced virus titers by approximately 2 log values. However, commencing treatment at 2 dpi or later resulted in virus titers in the treated group that were similar to those in the placebo group, indicating a lack of antiviral effect when treatment commenced at 2 dpi, at the time of symptom onset.

Analysis of the levels of major cytokines/chemokines in BALFs revealed that only early treatment, starting at 0 and 1 dpi, significantly decreased the levels of pro-inflammatory cytokines/chemokines (Figure 2B). This result suggests that the cytokine responses were consistent with the virus titers. Furthermore, histopathological analysis of the lungs of mice from various treatments using hematoxylin and eosin (HE) staining indicated that immediate administration of baloxavir prevented lung damage. Conversely, treatment at 1 dpi resulted in evident lung damage characterized by thickened alveolar septa, increased cellular accumulation, and reduced airspace compared to mock-infected mice. Therapy at 2 dpi or later resulted in substantial lung damage (Figure 2C).

Based on observations of body weight, clinical score, survival rate, virus titer, and lung histopathology, the therapeutic window for baloxavir in the mouse model of severe influenza appears to be approximately 1 day p.i.

### 3.2 Oclacitinib monotherapy offers protective effects in mice infected with the lethal influenza virus when administered at the mid-stage of infection

**FIGURE 3:**
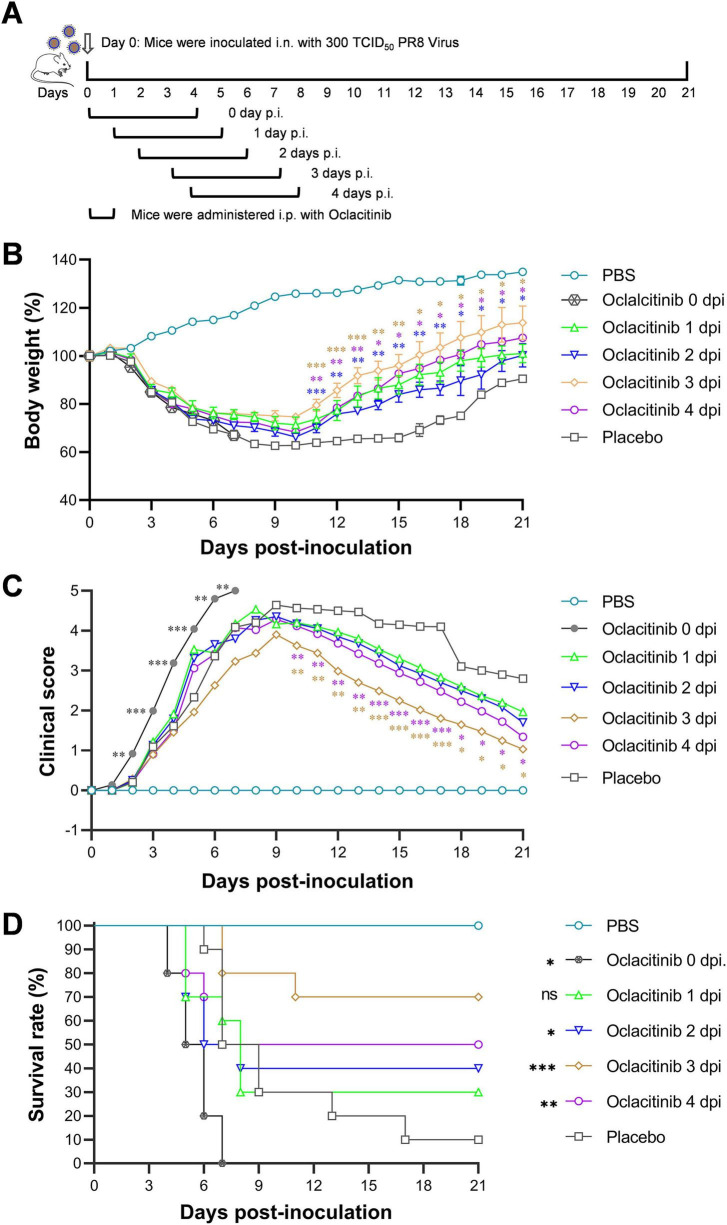
Evaluation of the therapeutic window for oclacitinib monotherapy in the treatment of lethal influenza in a murine model. **(A)** Diagram illustrating the experimental protocol for drug administration. Mice were infected with 300 TCID50 of the PR8 virus (10 mice per group) and subsequently treated with oclacitinib (20 mg/kg) at 0, 1, 2, 3, or 4 dpi, administered twice daily for five consecutive days in separate groups. **(B–D)** Body weight percentages **(B)**, clinical scores **(C)**, and survival rates **(D)** were monitored daily for 21 days. Statistical significance for panels **(B)** and **(C)** was assessed using two-way ANOVA, followed by Sidak’s post-test. Statistical significance for panel **(D)** was determined using a log-rank test. *p < 0.05, **p < 0.01, ***p < 0.001; ns indicates no significance.

An *in vivo* study was conducted to determine the therapeutic window of oclacitinib in 6-week-old BALB/c mice. Six groups of 10 mice were intranasally infected with 300 TCID50 of influenza virus. One group received a placebo, while the other 5 received 20 mg/kg/day oclacitinib starting at 0, 1, 2, 3, or 4 dpi. All treatments were administered twice daily for 5 days. An additional group was mock-infected with phosphate-buffered saline (PBS) and received no treatment. The mice were monitored for survival, body weight loss, and clinical scores over 21 days (Figure 3A).

As shown in Figure 3B, the body weight of mice treated with oclacitinib and placebo decreased upon influenza infection. Placebo-treated mice reached a minimum weight of 62.67% of their initial weight by 9 dpi. In contrast, the body weights of mice treated with oclacitinib starting at 1, 2, 3, and 4 dpi decreased to 71.40, 66.27, 74.66, and 68.30% of their initial body weights, respectively. Notably, immediate treatment led to a significant decline, with the weight falling to 67% at 7 dpi, resulting in the death of all treated mice that day.

Regarding the clinical scores, mice treated with oclacitinib at 3 dpi exhibited mild clinical symptoms, as evidenced by the clinical scores. At the peak of severity, the clinical score for placebo-treated mice was 4.64 (on day 9). In contrast, the clinical scores for mice treated with oclacitinib were 4.53 (on day 8), 4.35 (on day 9), 3.90 (on day 9), and 4.28 (on day 9) when oclacitinib treatment was initiated at 1–4 dpi Treatment of mice with oclacitinib immediately after viral infection (0 dpi) resulted in a rapid increase in clinical illness, reaching the highest score at 7 dpi, accompanied by 100% mortality (Figure 3C).

Mice treated with oclacitinib starting at 3 dpi had a survival rate of 70% (7/10), which was significantly higher (*p* < 0.01) than that of placebo-treated mice (10%, 1/10), representing the highest survival among all timings. Oclacitinib provided protection when administered at 2 (40%, 4/10), 3 (70%, 7/10), and 4 dpi (50%, 5/10). However, protection was minimal at 1 dpi (30%, 3/10) and none at 0 dpi, where all mice died within a week, indicating a worse outcome than the placebo (Figure 3D).

**FIGURE 4:**
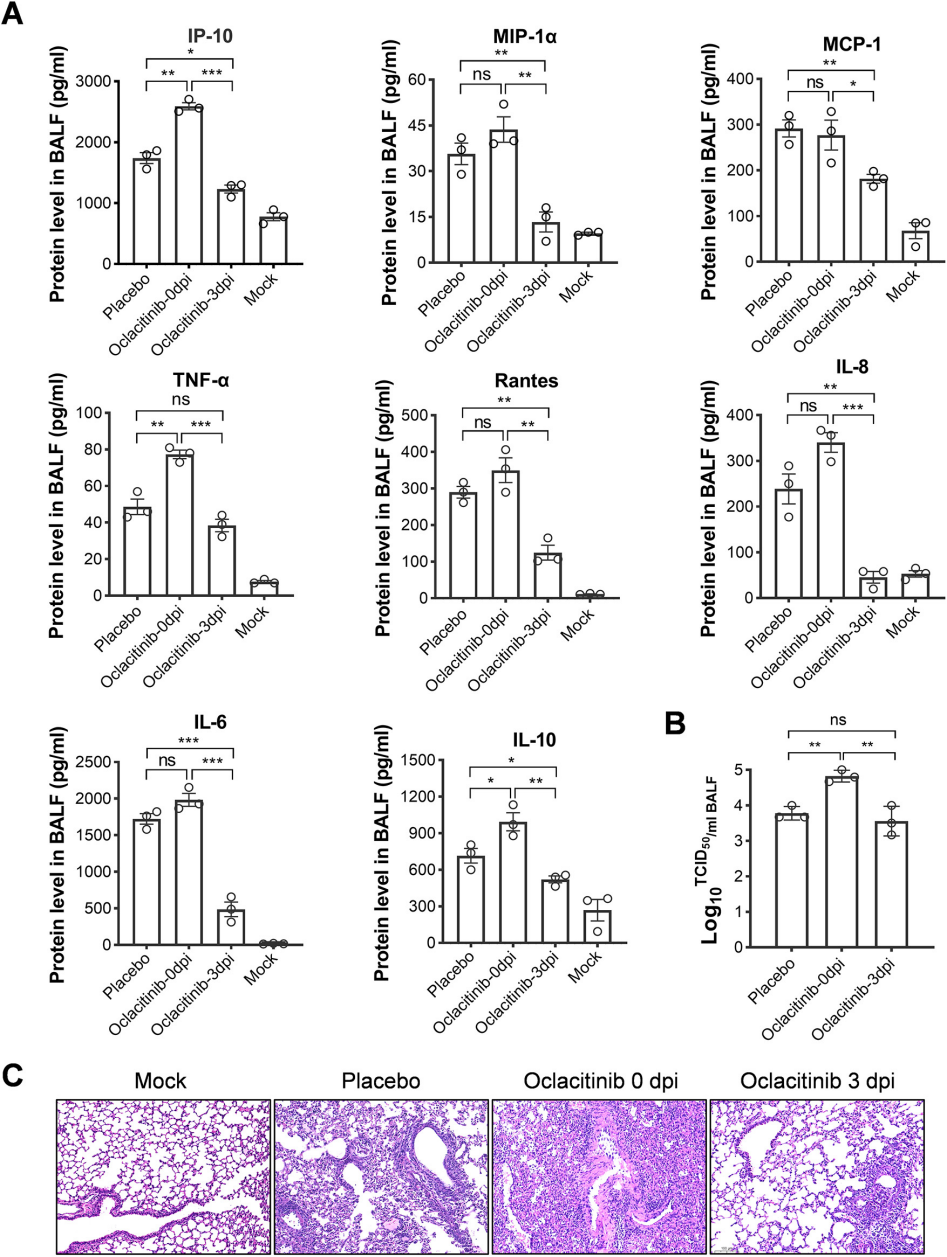
Assessment of virus production, cytokine/chemokine levels, and lung damage in mice infected with lethal influenza and treated with oclacitinib at various time points. Mice (*n* = 3 per group) was infected with 300 TCID50 of the PR8 virus and subsequently administered oclacitinib at a dose of 20 mg/kg at 0 and 3 dpi, with treatment occurring twice daily for five consecutive days. The control group received a placebo. An additional group of mice was mock-infected with phosphate-buffered saline (PBS). **(A)** Protein levels of pro-inflammatory cytokines and chemokines in BALFs from both oclacitinib- and placebo-treated groups (3 mice per group) were quantified by ELISA. **(B)** Viral titers in BALFs (3 mice per group), collected on day 7 p.i., were determined using a TCID50 assay with MDCK cells. **(C)** Hematoxylin and eosin staining of lung sections from oclacitinib-treated, placebo-treated, and normal control mice (3 mice per group) (scale bar: 100 μm). Data are expressed as mean ± standard error of the mean (SEM). Statistical significance in panels **(A,B)** was evaluated using an unpaired Student’s *t*-test, with the results denoted as follows: *p < 0.05, **p < 0.01, ***p < 0.001; ns indicates no significance.

Next, we evaluated the protein levels of eight pro-inflammatory cytokines and chemokines in the BALFs of mice infected with severe influenza. Surprisingly, BALF samples treated with oclacitinib starting at 0 dpi showed no significant changes in cytokine levels, with some, such as IP-10, IL-10, and TNF-α, actually increasing. In contrast, treatment with oclacitinib, initiated at 3 dpi, significantly reduced the levels of all measured cytokines, except for TNF-α, compared to the placebo group (*p* < 0.05) (Figure 4A). Early treatment with oclacitinib was associated with a higher viral titer (4.82 vs. 3.78), suggesting enhanced viral replication. However, treatment starting at 3 dpi showed no significant change in virus titer (3.56 vs. 3.78) (Figure 4B).

In this study, lung slices from mice were examined after HE staining following different oclacitinib treatments. Oclacitinib treatment starting at 0 dpi caused significant lung damage, with increased cell infiltration and reduced airspace compared to placebo. In contrast, starting treatment at 3 dpi resulted in mild lung damage, with intact lung structure and reduced inflammatory cell infiltration compared with placebo (Figure 4C).

Taken together, the results of our study on the use of oclacitinib as monotherapy for severe influenza in a mouse model indicate that the optimal therapeutic window lies in the mid-stage of the influenza infection. Effective treatment was observed when administered to mice at 2–4 dpi, with the most favorable outcomes occurring at 3 dpi. It is important to note that early treatment with oclacitinib should be avoided, as it can promote viral replication and exacerbate lung damage.

### 3.3 A combination of baloxavir and oclacitinib therapy extends the therapeutic window of antiviral and anti-inflammatory monotherapies

Clinical research suggests that the effective therapeutic window for antiviral baloxavir is less than 2 days during the early stages of influenza infection (Chan and Hui, 2023). In our current study using a mouse model of influenza, we found that a single dose of baloxavir was only effective when administered before 1 dpi. Conversely, the anti-inflammatory agent oclacitinib exhibited a therapeutic window from days 2 to 4 p.i. Although the therapeutic window of oclacitinib is broader than that of baloxavir, both agents present significant challenges: baloxavir demonstrates reduced efficacy in later stages of infection, whereas oclacitinib poses the risk of lung damage if administered too early. Additionally, patient variability further complicates the determination of the optimal therapeutic window. Based on these observations, we hypothesized that combined therapy with both agents may mitigate the limitations of individual treatments.

To test our hypothesis, we evaluated combined treatment with baloxavir and oclacitinib in a mouse model of influenza. Six groups of BALB/c mice (10 mice each) were infected with the influenza virus. One group received a placebo, while the other groups received oral baloxavir (10 mg/kg/d) and oclacitinib (20 mg/kg/d) starting on different dpi. All mice received baloxavir on the first day, and oclacitinib was administered twice daily for 5 days. The mock group was treated with PBS and served as the control. The mice were monitored daily for 21 days for survival, weight loss, and clinical scores.

**FIGURE 5:**
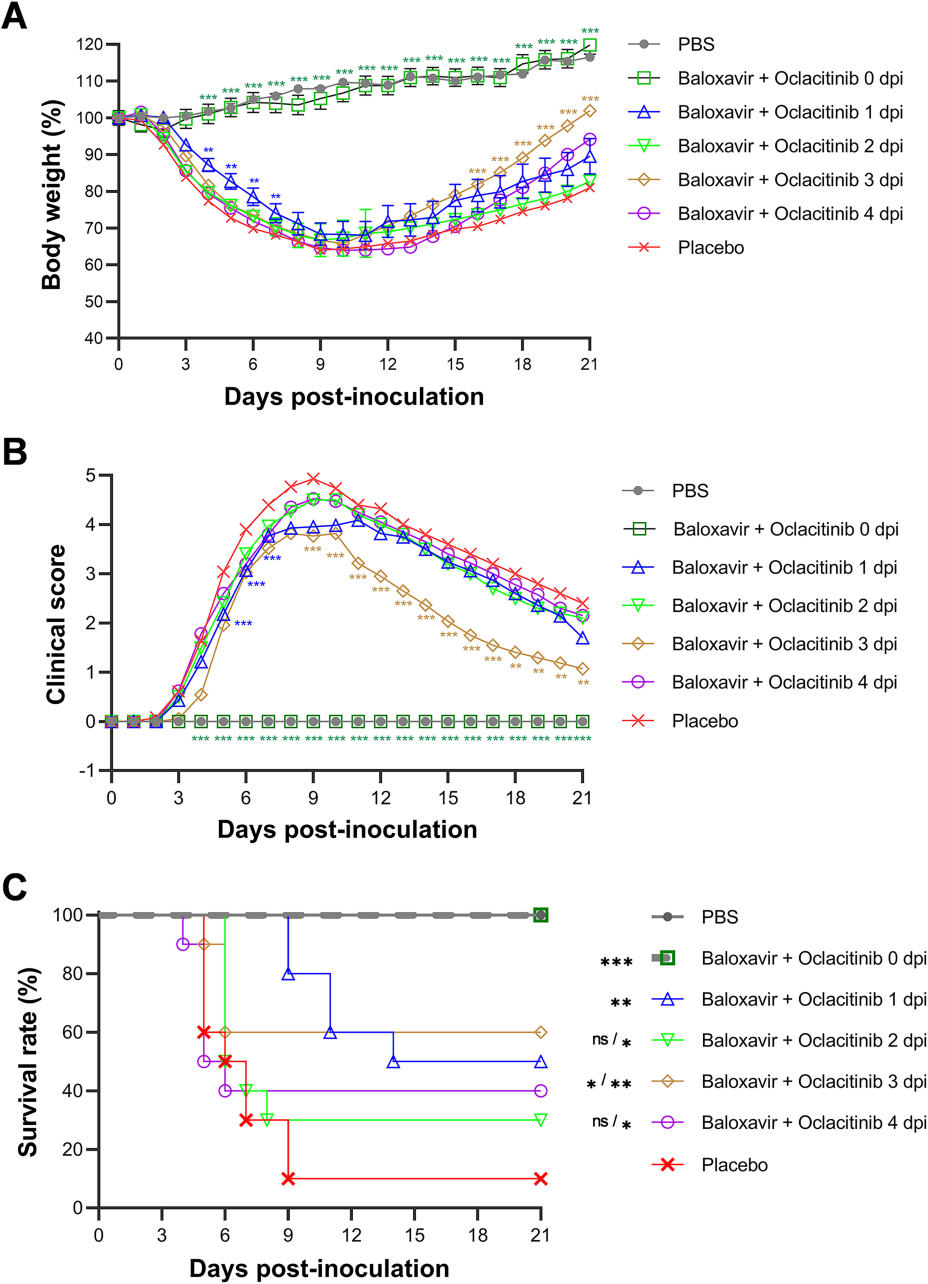
Efficacy of combination therapy with baloxavir and oclacitinib in treating mice infected with a lethal strain of influenza virus. Six groups of mice (*n* = 10 per group) were inoculated with 300 TCID50 PR8 virus. Subsequently, the subjects were treated with baloxavir at a dosage of 10 mg/kg/day and oclacitinib at 20 mg/kg/day at 0, 1, 2, 3, or 4 dpi or were administered a placebo. All mice that were treated with baloxavir received only a single dose on the initial day of treatment. In contrast, all mice treated with oclacitinib were administered the medication twice daily for five consecutive days. Additionally, a separate group of 10 mice inoculated with an equal volume of PBS served as the mock-infected controls. Variables, including body weight percentages **(A)**, clinical scores **(B)**, and survival rates **(C)**, were monitored daily for 21 days. The statistical significance of the data presented in panels **(A,B)** was computed using a two-way ANOVA accompanied by Sidak’s post-test. For panel **(C)**, significance was determined using the log-rank test. The results are denoted as follows: *p < 0.05, **p < 0.01, ***p < 0.001, with “ns” indicating no statistical significance.

In the context of combination therapy, both mock-infected mice and those receiving treatment at 0 dpi exhibited progressive gain in body weight. In contrast, mice that received either placebo or the combined pharmacological agents from 1 dpi experienced a decline in body weight, reaching a nadir at approximately 10 dpi, followed by a recovery phase. Notably, mice undergoing combination therapy demonstrated a more accelerated recovery in body weight than those receiving the placebo (Figure 5A).

Regarding clinical scores, mock-infected mice and those initiating combination therapy at 0 dpi showed no adverse health effects. Furthermore, mice receiving combination therapy showed milder symptoms, as indicated by lower peak clinical scores, than those treated with placebo. Consistent with the findings from the baloxavir monotherapy experiment, mice treated with both agents from 0 dpi onward exhibited no signs of illness, closely resembling the health status of mock-infected mice (Figure 5B).

Importantly, all mice receiving the combined drugs from 0 dpi survived exposure to a lethal dose of the influenza virus (10/10, *p* < 0.001). The survival rates of mice treated with combination therapy initiated at 1 and 3 dpi were 50% (5/10) and 60% (6/10), respectively, both of which demonstrated statistically significant differences (*p* < 0.001) compared with the placebo group. However, the survival rate of mice treated at 2 and 4 dpi did not reach statistical significance (*p* < 0.01) when comparing the entire disease course, but did when comparing the last 18 days of the disease. Their combination therapy conferred protection against lethal influenza virus infection in 30 and 40% of mice (Figure 5C).

**FIGURE 6:**
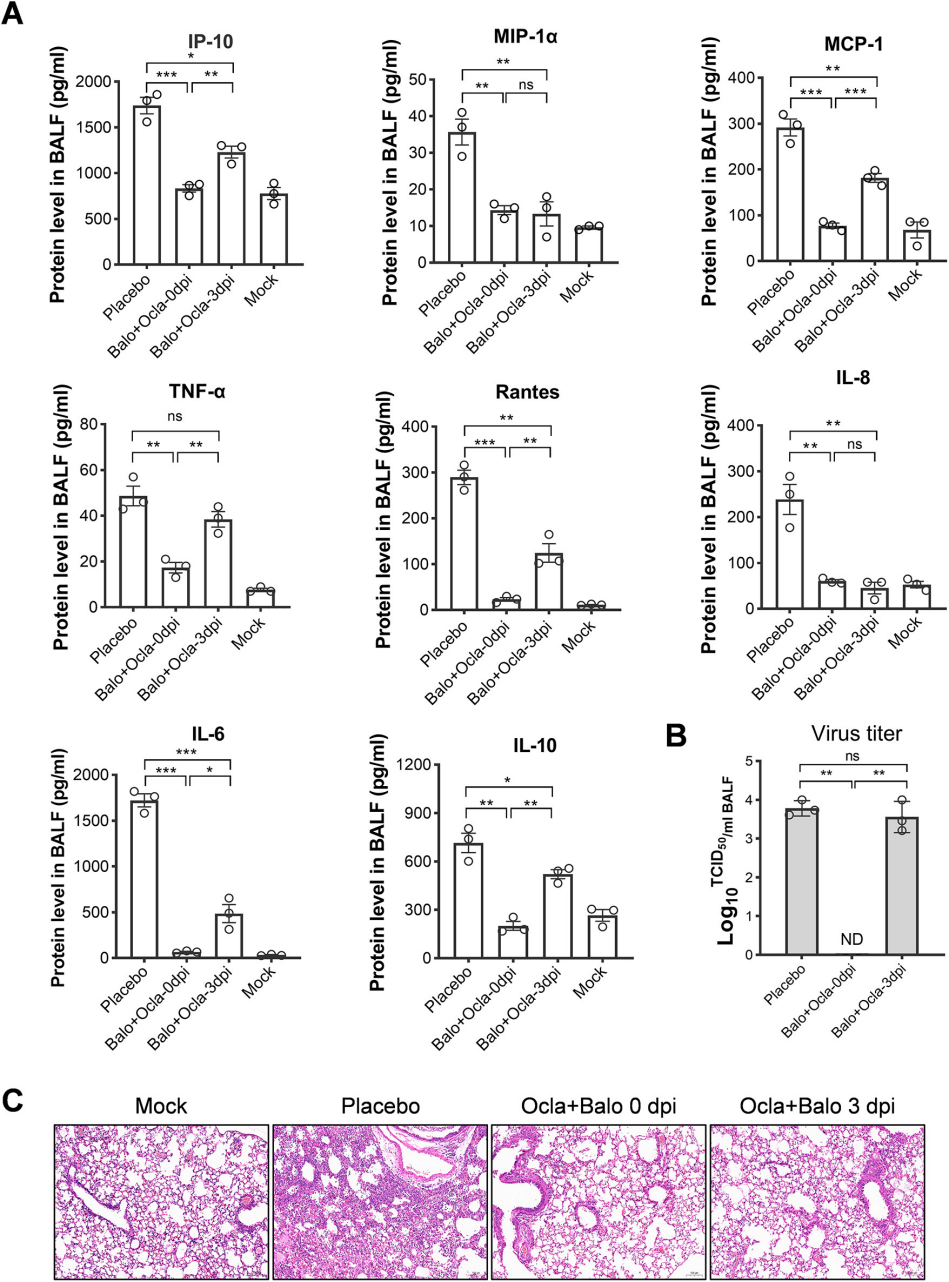
Evaluation of viral production, cytokine/chemokine levels and lung lesions in mice infected with lethal influenza and administered a combination of baloxavir and oclacitinib. Two groups of mice (*n* = 3 per group) were infected with 300 TCID50 of PR8 virus and subsequently treated with baloxavir (10 mg/kg) and oclacitinib (20 mg/kg per day) at days 0 and 3 post-infection (dpi). All mice in the baloxavir group received a single dose, whereas those receiving oclacitinib were treated twice daily for five consecutive days. A control group of mice was infected with 300 TCID50 PR8 virus and treated with a placebo on day 0, while the other group was mock-infected with PBS. All mice were euthanized with cervical dislocation on day 7 p.i., and their BALFs were collected for the measurement of cytokine production and progeny viral titers. **(A)** Viral titers in BALFs collected at 7 dpi were determined using the TCID50 assay on MDCK cells (3 mice per group). **(B)** The protein concentrations of pro-inflammatory cytokines and chemokines in BALFs obtained on day 7 p.i. from groups receiving combination therapy initiated at various time points were quantified using ELISA. **(C)** Hematoxylin and eosin staining was performed on lung tissues from mice treated with baloxavir and oclacitinib at 0 dpi, and from those treated with baloxavir and oclacitinib at 3 dpi, as well as from placebo-treated and normal control mice (3 mice per group) (scale bar: 100 μm). Data are presented as mean values ± standard error of the mean (SEM). Statistical significance in both **(A,B)** was evaluated using an unpaired Student’s *t*-test. *p < 0.05, **p < 0.01, ***p < 0.001; ns indicates no significance.

**FIGURE 7:**
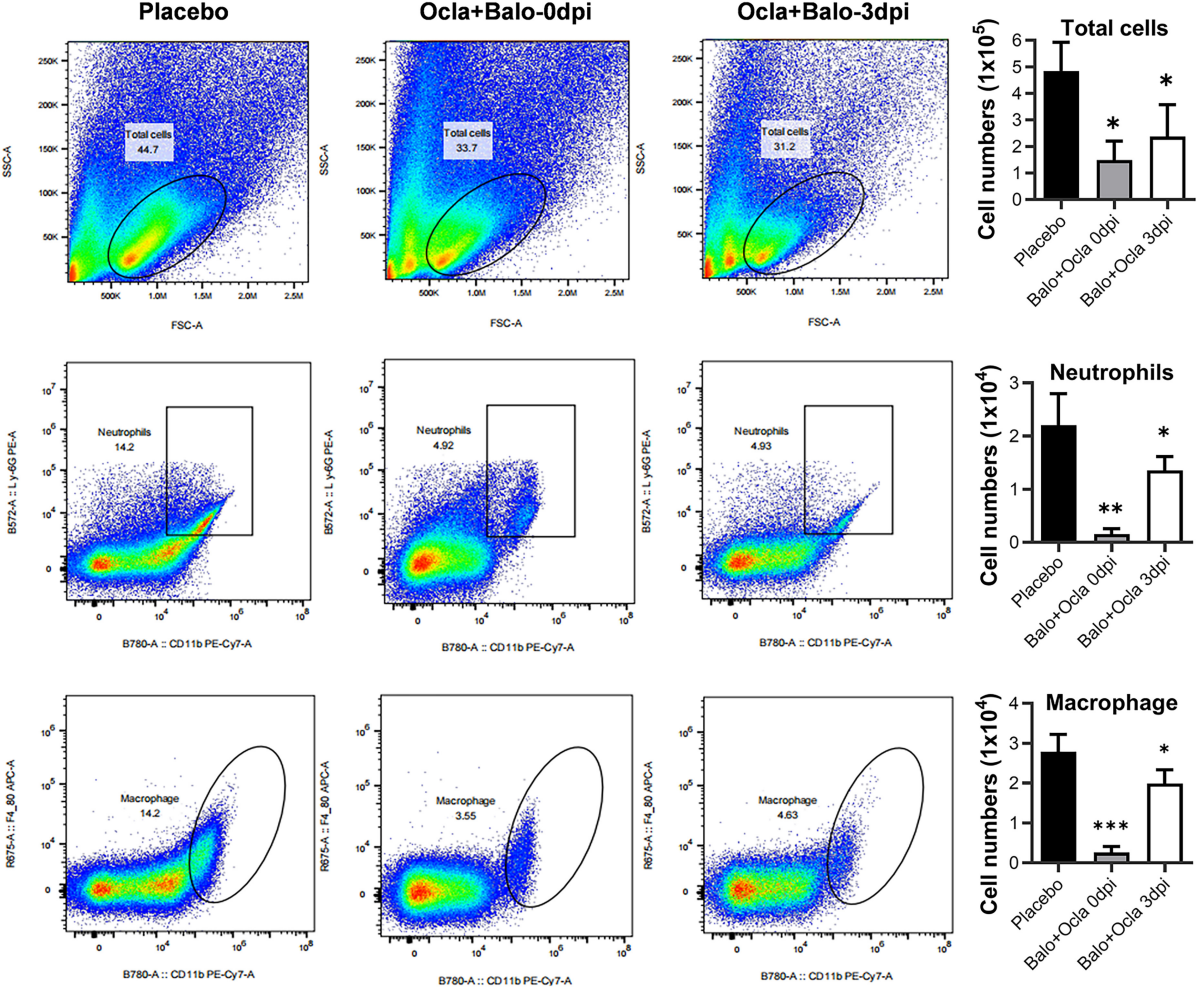
Effects of combination therapy utilizing baloxavir and oclacitinib on the suppression of neutrophil and macrophage infiltration in mice infected with a lethal strain of the influenza virus. Three groups of mice (*n* = 3 per group) were infected with 300 TCID50/50 μL of the PR8 virus. Two groups received treatment comprising baloxavir (10 mg/kg/day) and oclacitinib (20 mg/kg/day), initiated on day 0 and day 3 post-infection, respectively. Baloxavir was administered as a single dose, whereas oclacitinib was administered twice daily for five consecutive days. A third group served as a control and received a placebo. On day 7 post-infection, all mice were euthanized, and their tracheas were isolated and injected with 1 mL of phosphate-buffered saline (PBS). The thoraxes of each mouse were gently massaged, followed by aspiration of the bronchoalveolar lavage fluid (BALF), which was subsequently analyzed via flow cytometry. The total cell count and the counts of neutrophils and macrophages in the BALFs were determined and compared between the combination therapies initiated at day 0 and 3 post-infection and the placebo groups. Results are presented as mean values ± standard error of the mean (SEM), with statistical significance indicated as *p < 0.05, **p < 0.01, and ***p < 0.001.

In addition, we evaluated the cytokine levels and virus titers in the BALFs of mice receiving combination therapy on day 7 p.i. Our preliminary results demonstrated that early treatment (0 dpi) significantly reduced proinflammatory cytokine levels (Figure 6A) and was accompanied by lower virus titers (Figure 6B). In contrast, treating infected mice during the mid-stage (e.g., 3 dpi) resulted in relatively low levels of proinflammatory cytokines, whereas virus titers remained high (Figures 6A,B). In alignment with the cytokine levels, lung tissue pathology in mice receiving combination therapy at both 0 and 3 dpi showed markedly milder lesions compared to those treated with placebo (Figure 6C). Furthermore, flow cytometry analysis indicated that combination therapy at both time points significantly decreased neutrophil and macrophage infiltration in BALFs, which are recognized contributors to acute lung injury associated with influenza pneumonia (Figure 7). In conclusion, combination therapy with baloxavir and oclacitinib significantly extended the treatment window for managing severe influenza virus infections, increasing it to 5 days. This extended duration surpasses that of antiviral monotherapy with baloxavir, which is limited to 1 day, and anti-inflammatory monotherapy with oclacitinib, which is effective for 3 days.

## 4 Discussion

Despite the presence of various antiviral medications for influenza virus infections, managing severe influenza poses a considerable challenge. Several factors contributed to this difficulty. Primarily, the rapid replication of highly pathogenic viral strains within the pulmonary systems of patients significantly shortens the therapeutic window for antiviral intervention, which is typically limited to only 1–2 days. Should the opportunity for antiviral treatment be missed, elevated viral titers may trigger cytokine storms, potentially resulting in severe pneumonia and acute respiratory distress syndrome (ARDS).

Due to the limited administration window, direct-acting antiviral (DAA) medications typically exhibit reduced efficacy in treating influenza virus infections when administered later in the course of the illness. Baloxavir and oseltamivir should be administered within a short timeframe (e.g., 48 h) of symptom onset to optimize therapeutic outcomes (Wutzler, 1997; O’Hanlon and Shaw, 2019; Chan and Hui, 2023). In line with previous studies, we observed that a single dose of baloxavir was therapeutically effective when administered within 24 h of infection with a lethal dose of the influenza virus (Figure 3). The advantages of early antiviral intervention align with treatment protocols for other viral diseases, including acute herpes zoster (Wutzler, 1997) and HIV infections (Watson et al., 1997). However, early administration of antiviral therapies remains critical for managing severe respiratory viral infections, such as influenza and coronaviruses, as demonstrated in the historical outbreaks of H5N1, 2009 H1N1 (Dunning et al., 2014), and SARS-CoV-2 (Li et al., 2020; McCullough, 2020).

The early administration of anti-inflammatory agents appears to have adverse effects in cases of influenza. Our previous research indicated that simultaneous administration of ponatinib during influenza virus infection in murine models does not reduce cytokine levels during the initial phase of disease; rather, it facilitates viral proliferation and delays viral clearance (Chen et al., 2019). The findings from the current study further substantiate that delayed administration of oclacitinib 3 days post-influenza virus infection affords greater protection than early intervention. The optimal therapeutic window occurs during the mid-stage of infection (2–4 dpi). Moreover, it is essential to note that early treatment with oclacitinib should be avoided, as it has been shown to enhance viral replication and worsen lung damage. Oclacitinib, a JAK inhibitor, has been approved by the FDA for the treatment of dermatitis in dogs and has been demonstrated to be effective in cats (Ortalda et al., 2015; Marsella et al., 2023). Therefore, oclacitinib has the potential for clinical use in cases of inflammation associated with influenza virus infection, and caution should be exercised regarding its timing of administration.

In our study, administration of oclacitinib at the mid-stage of the disease reduced cytokine and chemokine levels in lung tissue, including IP-10, MIP-1α, MCP-1, TNF-α, Rantes, IL-8, IL-6, and IL-10 (Figure 4A). However, the progeny virus titer in the lung tissues of oclacitinib-treated mice initiated at 3 dpi remained unaffected (Figure 4B). These findings suggest that oclacitinib inhibits the JAK-STAT signaling pathway in the host rather than directly interfering with progeny virus replication.

The window for antiviral treatment is short and should be initiated early; however, patients often miss this timing. In contrast, the therapeutic window for the anti-inflammatory medication oclacitinib is during the mid-stage of influenza infection. Early administration of oclacitinib can cause significant lung damage. We hypothesized that combining antivirals and anti-inflammatory agents may reduce the drawbacks of using either type alone, potentially improving clinical outcomes.

In combination therapy, the therapeutic window for baloxavir and oclacitinib was extended to 5 days (0–4 dpi), longer than that for baloxavir alone (2 days, 0–1 dpi) or oclacitinib alone (3 days, 2–4 dpi). Notably, the efficacy of administration at 2 and 4 dpi was marginally lower than that at 0, 1, and 3 dpi (Figure 5C). Therefore, the overall therapeutic effect appears to lie between additive and synergistic effects, indicating that managing acute respiratory viral infections, particularly severe influenza, continues to pose significant challenges, even when effective antiviral and anti-inflammatory agents are available. Thus, exploration of new drugs with distinct therapeutic mechanisms or new drug combinations may provide a broader therapeutic window and enhance the potential for effective treatment outcomes.

In summary, we assessed the therapeutic window of baloxavir and oclacitinib in a BALB/c mouse model of influenza. Our findings confirmed that baloxavir demonstrates antiviral efficacy primarily in the early stages of influenza by reducing viral titers, thereby mitigating lung pathology. Conversely, oclacitinib proved beneficial during the mid-stages of influenza by lowering levels of various pro-inflammatory cytokines and chemokines. However, it had a minimal impact on viral replication, ultimately leading to reduced lung injury.

A key consideration in this study was the rationale for the selected dosages of baloxavir and oclacitinib. These doses were based on prior optimization studies in our laboratory (Yu et al., 2024), which showed optimal therapeutic efficacy against influenza in a murine model while maintaining safety. The main objective was to test the hypothesis that combining an antiviral with a JAK inhibitor may enhance the therapeutic window using established effective doses for each treatment. Although other concentrations could be explored, this proof-of-concept study primarily aimed to validate the combination strategy. Further dose-ranging studies in preclinical models will be necessary to optimize this pairing and ensure the safety and efficacy of the dosages for human use.

The findings from our single-dose model underscore the significance of timed combination therapy. Future research on multi-dose regimens of baloxavir would better reflect the management of severe cases in human clinical practice and could potentially broaden the therapeutic window. Furthermore, this study did not evaluate the emergence of baloxavir-resistant variants of the influenza virus. Considering that immunosuppression may heighten the risk of antiviral resistance, future investigations that incorporate viral sequencing from treated subjects, particularly those receiving immunomodulatory treatment, will be crucial for a comprehensive assessment of the long-term efficacy of this combination strategy.

When examining the limitations of both monotherapies, it became clear that combination therapy with baloxavir and oclacitinib extended the therapeutic window and reduced the risk of increased viral replication associated with early oclacitinib use. Notably, both humans and mice have similar timelines for viral replication and clearance (Bender and Small, 1993; Huang et al., 2014; Georgel et al., 2019), although these timelines can vary based on several factors. The diversity among patients with influenza, including age, genetic background, overall health status, and level of exposure to the virus, complicates the identification of an optimal therapeutic window for antiviral or anti-inflammatory treatments.

In conclusion, our findings suggest that a combined therapeutic approach using both an antiviral agent and an anti-inflammatory drug is more effective than either medication alone. Although the outcomes of the current study regarding this combined treatment may not be ideal, there is significant potential to enhance treatment effectiveness through further investigation of various drug combinations, particularly those that employ multiple anti-influenza mechanisms targeting both antiviral and anti-inflammatory responses. This is particularly pertinent for managing severe cases of influenza.

## Data Availability

The original contributions presented in the study are included in the article/supplementary material, further inquiries can be directed to the corresponding authors.
